# Supplementary material for: The Small RNA Universe of Capitella teleta
Source: Front Mol Biosci. 2022 Feb 25;9:802814. doi: 10.3389/fmolb.2022.802814 (PMC8915122; doi:10.3389/fmolb.2022.802814)
Supplement: Supplementary file 1 [file DataSheet1.ZIP › Supplement/known/CAPTEscaffold_156_11571.pdf]

Provisional ID	:	CAPTEscaffold_156_11571
Score total	:	8870.6
Score for star read(s)	:	3.9
Score for read counts	:	8859.9
Score for mfe	:	2.2
Score for randfold	:	1.6
Score for cons. seed	:	3
Total read count	:	17390
Mature read count	:	17348
Loop read count	:	0
Star read count	:	42

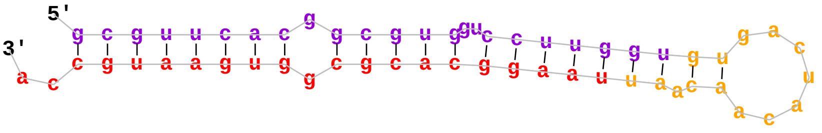

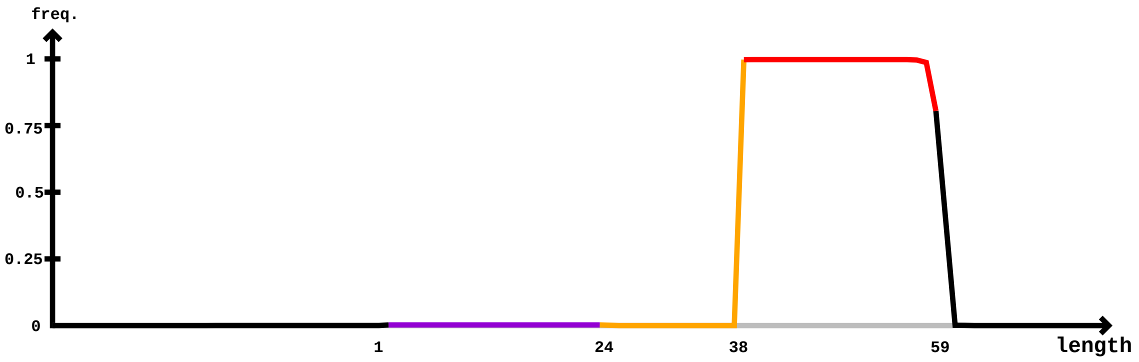

Star Mature

```
5'- gcaugcagaggacguuguucuggcgacuucuuggcguucacggcgugguccuuggugugacuacaacaauuaaggcacgcggugaaugccaagacgaccgccgcugcc -3'   obs
    gcaugcagaggacguuguucuggcgacuucuuggcguucacggcgugguccuuggugugacuacaacaauuaaggcacgcggugaaugccaagacgaccgccgcugcc        exp
    gcaugcagaggacguuguucuggcgacuucuuggcguucacggcgugguccuuggugugacuacaacaauuaaggcacgcggugaaugccaagacgaccgccgcugcc        known
    ....((((((((...)))))((((...((((((((((((.(((((..((((((((.......)).))))))))))).))))))))))))....)))).)))).      reads   mm   sample
    ...............uguucuggcgacuucuug...........................................................................      4       0    seq
    .................................gcguucacggcgugguccuugg.....................................................      1       0    seq
    .................................gcguucacggcgugguccuuAgu....................................................      1       1    seq
    .................................gcguucacggcgugguAcuuggu....................................................      1       1    seq
    .................................gcguucacggcgugguccuuggu....................................................      37      0    seq
    .................................gcguucacggcgugguccuugguU...................................................      2       1    seq
    ......................................................................Aaaggcacgcggugaaug....................      1       1    seq
    ......................................................................uaaggcacgcggugaaug....................      26      0    seq
    ......................................................................uaaggcacgcggugaaugc...................      159     0    seq
    ......................................................................uaaggcacgcggugaaugU...................      3       1    seq
    ......................................................................Aaaggcacgcggugaaugc...................      2       1    seq
    ......................................................................uaaggcCcgcggugaaugcc..................      1       1    seq
    ......................................................................uaaggcacgcggugaaAgcc..................      5       1    seq
    ......................................................................uaaggcacgcggugUaugcc..................      1       1    seq
    ......................................................................uaaggcacgcggugaGugcc..................      1       1    seq
    ......................................................................uaaggcacgcggugaaugcc..................      3088    0    seq
    ......................................................................uaaggcGcgcggugaaugcc..................      11      1    seq
    ......................................................................uaaggcacgcAgugaaugcc..................      4       1    seq
    ......................................................................Aaaggcacgcggugaaugcc..................      19      1    seq
    ......................................................................uaaggcacgcggugaaugcG..................      1       1    seq
    ......................................................................uaaggcacgcggugaaugcU..................      3       1    seq
    ......................................................................uaaAgcacgcggugaaugcc..................      1       1    seq
    ......................................................................uaaggcaUgcggugaaugcc..................      1       1    seq
    ......................................................................uaaCgcacgcggugaaugcc..................      1       1    seq
    ......................................................................uaaggAacgcggugaaugcc..................      1       1    seq
    ......................................................................uaaggcacgcgguAaaugcc..................      7       1    seq
    ......................................................................uGaggcacgcggugaaugcc..................      1       1    seq
    ......................................................................uaaggcacgcggAgaaugcc..................      2       1    seq
    ......................................................................Gaaggcacgcggugaaugcc..................      6       1    seq
    ......................................................................uUaggcacgcggugaaugcc..................      1       1    seq
    ......................................................................Naaggcacgcggugaaugcc..................      1       1    seq
    ......................................................................uaaggcacgcgAugaaugcc..................      1       1    seq
```

```
                                  Star                                  Mature
gcaugcagaggacguuguucuggcgacuucuuggcguucacggcgugguccuuggugugacuacaacaauuaaggcacgcggugaaugccaagacgaccgccgcugcc
......................................................................uaaggcacgcggugaaugcA..................   6      1   seq
......................................................................uaGggcacgcggugaaugcca.................   4      1   seq
......................................................................uaaggcacgcggugaaugGca.................   1      1   seq
......................................................................uaUggcacgcggugaaugcca.................   2      1   seq
......................................................................uaaggcacgcggugaaugccU.................   204    1   seq
......................................................................uGaggcacgcggugaaugcca.................   9      1   seq
......................................................................uaaggUacgcggugaaugcca.................   2      1   seq
......................................................................uaaggcCcgcggugaaugcca.................   1      1   seq
......................................................................uaaUgcacgcggugaaugcca.................   1      1   seq
......................................................................uaaggcacgcggugaauUcca.................   1      1   seq
......................................................................uaagAcacgcggugaaugcca.................   1      1   seq
......................................................................uaaggcacgcggugaaugUca.................   1      1   seq
......................................................................uaaggGacgcggugaaugcca.................   1      1   seq
......................................................................uaaggcacgcggugaaGgcca.................   2      1   seq
......................................................................uaaggcacgcggugaaugcAa.................   13     1   seq
......................................................................uaaggcacgcggugaaugcUa.................   10     1   seq
......................................................................uaaggcacgcggAgaaugcca.................   11     1   seq
......................................................................uaaggcacgGggugaaugcca.................   1      1   seq
......................................................................uaaggcacAcggugaaugcca.................   3      1   seq
......................................................................uaaggcacgcUgugaaugcca.................   3      1   seq
......................................................................uaaggcacgAggugaaugcca.................   4      1   seq
......................................................................uaaggcacgcgAugaaugcca.................   4      1   seq
......................................................................uaaggcacgcggugaauCcca.................   1      1   seq
......................................................................uaaggcacgcggugaaugcca.................   13369  0   seq
......................................................................uaaggcacgcggugaaCgcca.................   4      1   seq
......................................................................uaaggcacgUggugaaugcca.................   2      1   seq
......................................................................uaaggcaAgcggugaaugcca.................   2      1   seq
......................................................................uaaggAacgcggugaaugcca.................   1      1   seq
......................................................................uaaggcacgcggugCaugcca.................   1      1   seq
......................................................................uaaggcacgcggugaauAcca.................   5      1   seq
......................................................................uaaggcacgcggugaUugcca.................   1      1   seq
......................................................................uaaggcacgcggugaaugAca.................   3      1   seq
......................................................................uaaggcacgcggugaGugcca.................   6      1   seq
......................................................................Caaggcacgcggugaaugcca.................   3      1   seq
......................................................................uaaggcacgcggugaaugccC.................   1      1   seq
......................................................................uaaggcacCcggugaaugcca.................   3      1   seq
......................................................................uaaAgcacgcggugaaugcca.................   1      1   seq
......................................................................uaaggcacgcggugaaAgcca.................   10     1   seq
......................................................................uaaggcacgcggugUaugcca.................   2      1   seq
......................................................................uaaggcacgcggugaaugccG.................   25     1   seq
......................................................................uaaggcacgcCgugaaugcca.................   2      1   seq
......................................................................Aaaggcacgcggugaaugcca.................   69     1   seq
......................................................................uaaggcacgcgguUaaugcca.................   1      1   seq
......................................................................Gaaggcacgcggugaaugcca.................   10     1   seq
......................................................................uaaggcacgcgguAaaugcca.................   23     1   seq
......................................................................Naaggcacgcggugaaugcca.................   3      1   seq
......................................................................uaaggcGcgcggugaaugcca.................   6      1   seq
......................................................................uaagCcacgcggugaaugcca.................   2      1   seq
......................................................................uaaggcaUgcggugaaugcca.................   5      1   seq
......................................................................uaaggcacgcggCgaaugcca.................   1      1   seq
......................................................................uaaggcacgcAgugaaugcca.................   25     1   seq
......................................................................uaaggcacgcgguCaaugcca.................   3      1   seq
......................................................................uaaggcacUcggugaaugcca.................   1      1   seq
......................................................................uaaggcUcgcggugaaugcca.................   1      1   seq
......................................................................uaaggcacgcgUugaaugcca.................   1      1   seq
......................................................................uaaggcacgcggGgaaugcca.................   1      1   seq
......................................................................uaaggcacgcggugaaugccaU................   92     1   seq
......................................................................uaaggcacgcggugaaugccaC................   2      1   seq
......................................................................uaaggcacgcggugaaugccaa................   7      0   seq
......................................................................uaaggcacgcggugaaugccaaA...............   4      1   seq
......................................................................uaaggcacgcggugaaugccaaAa..............   13     1   seq
.......................................................................aaggcacgcggugaaugcc..................   1      0   seq
.......................................................................aaggcacgcggugaaugcca.................   1      0   seq
.......................................................................Uaggcacgcggugaaugcca.................   1      1   seq
```
